# Oxidative Damage of DNA as Early Marker of Alzheimer’s Disease

**DOI:** 10.3390/ijms20246136

**Published:** 2019-12-05

**Authors:** Carmen Peña-Bautista, Tania Tirle, Marina López-Nogueroles, Máximo Vento, Miguel Baquero, Consuelo Cháfer-Pericás

**Affiliations:** 1 Neonatal Research Unit, Health Research Institute La Fe, 46026 Valencia, Spain; carpebau@alumni.uv.es (C.P.-B.); tatir@alumni.uv.es (T.T.); maximo.vento@uv.es (M.V.); 2 Analytical Unit Platform, Health Research Institute La Fe, 46026 Valencia, Spain; marina_lopez@iislafe.es; 3 Neurology Unit, University and Polytechnic Hospital La Fe, 46026 Valencia, Spain; miquelbaquero@gmail.com

**Keywords:** DNA, protein, oxidative stress, urine, mass spectrometry, mild cognitive impairment

## Abstract

Alzheimer’s Disease (AD) is the most common cause of dementia, and its characteristic histopathological hallmarks are neurofibrillary tangles and senile plaques. Among involved mechanisms, oxidative stress plays an important role in damaging cell components (e.g., proteins, nucleic acids). In this study, different oxidized products of proteins and DNA were determined in the urine samples from mild cognitive impairment due to AD patients (*n* = 53) and healthy controls (*n* = 27) by means of ultra-performance liquid chromatography-tandem mass spectrometry analysis. A multivariate model developed by partial least squares generated a diagnostic model for AD with an AUC-ROC (area under the curve-receiver operating characteristic) of 0.843. From the studied analytes, 8-OHdG (8-hydroxy-2’-deoxyguanosine) and the ratio 8-OHdG/2dG (2’-deoxyguanosine) were able to distinguish between AD and healthy participants, showing statistically significant differences between groups, postulating DNA oxidation as a molecular pathway involved in early AD.

## 1. Introduction

Alzheimer’s disease (AD) is a progressive neurodegenerative disease characterized by neuronal cell loss and accumulation of proteins forming neurofibrillary tangles (NFT) and amyloid fibers in the senile plaques. The main clinical symptoms are cognitive impairment, memory loss, and physical deterioration [1]. Regarding the pathological pathways that may be involved in AD, oxidative stress plays an important role since it is related to neuronal degeneration [2,3]. In fact, these conditions could lead to oxidative damage of cellular components (lipids, proteins, nucleic acids), and so damaging cellular functions [4,5]. Therefore, the accumulation of these oxidized biomolecules could be involved in the development of AD [4]. In spite of the high evidence of protein impairment in AD, few studies have focused on the oxidative damage to nitrogenated compounds [6].

**Figure 1:**
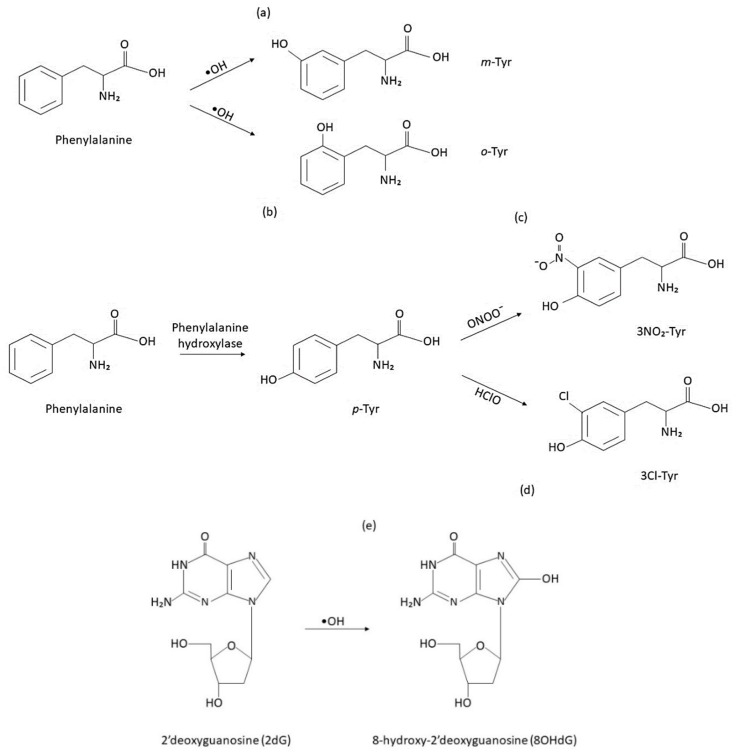
Oxidative modifications to proteins and DNA resulting in oxidized biomarkers: m-Tyr (meta-tyrosine) (**a**), o-Tyr (ortho-tyrosine) (**b**), 3-NO2-Tyr (3-nitrotyrosine) (**c**), 3-Cl-Tyr (3-chlorotyrosine) (**d**), and 8-OHdG (8-hydroxy-2’-deoxyguanosine) (**e**).

There is large evidence showing that the brain is particularly vulnerable to oxidative stress because of its low levels of antioxidants (glutathione) and high metabolic utilization of oxygen [2,7]. Specifically, this damage is related to an increase in the levels of lipid peroxidation compounds, oxidized proteins (3-nitrotyrosine (3-NO2-Tyr), protein-bound carbonyls) (Figure 1), and nucleic acids (8-hydroxy-2’-deoxyguanosine (8-OHdG), 8-OH-guanosine) [2,8,9,10]. Studies have reported that nitration and carbonylation can alter protein functionality. Also, the increased levels of 3-NO_2_-Tyr have been linked to inflammation and apoptosis in pathological conditions [11,12]. Other oxidation products are 3-chlorotyrosine (3-Cl-Tyr) (Figure 1d), and the isomers ortho-tyrosine (o-Tyr) and meta-tyrosine (m-Tyr) (Figure 1a,b). All of these products are considered markers of nitrosative or oxidative damage to proteins [13]. Regarding DNA oxidative damage, 8-OHdG results from the hydroxylation of the free base 2′-deoxyguanosine (2dG) (Figure 1e), and it is a widely accepted biomarker of DNA damage [5,14].

Several studies have measured the levels of these products in biological fluids and tissues, such as blood, urine, cerebrospinal fluid (CSF), and brain [1,7,8,9,10,15,16,17]. Regarding the analytical methods, they are mainly based on high-performance liquid chromatography (HPLC) coupled with electrochemical detection (EC) or mass spectrometry, enzyme-linked immunosorbent assay (ELISA) and other immunoassays, 2,4-dinitrophenylhydrazide (DNPH) assay, and spectrophotometry. Several groups reported increased levels of 3-NO_2_-Tyr in CSF [8,10,15,16] and plasma [10,17] from patients with mild cognitive impairment due to AD (MCI-AD) compared to control subjects. Likewise, protein carbonyls determined in peripheral blood by immunoassays [17] and spectrophotometry showed increased levels in AD patients [1,9]. For DNA oxidation, data showed an increase in the plasma levels of 8-OHdG in AD patients [18,19], suggesting that this oxidized product might be used as an AD diagnosis marker.

To our knowledge, this is the first study carried out to determine nine proteins and DNA oxidative damage biomarkers in urine samples from well-defined early AD patients rather than frank AD patients, using a validated analytical method based on liquid chromatography coupled to tandem mass spectrometry. The aim of this study was the assessment of oxidative stress damage to protein and DNA as potential early mechanisms in AD development. For this, a panel of biomarkers for DNA oxidation (8-OHdG/2-dG), protein nitration (3-NO_2_-Tyr/p-Tyr), oxidation (m-Tyr/Phe and o-Tyr/Phe), and chlorination (3Cl-Tyr/p-Tyr) were determined.

## 2. Results

### 2.1. Demographic and Clinical Variables

The participants of the study were classified into control (*n* = 27) and case (*n* = 53) groups. The criteria used to classify the participants were based on the following variables: neuropsychological tests, such as repeatable battery for the assessment of neuropsychological status (RBANS) [20], functional activities questionnaire (FAQ) [21], clinical dementia rating (CDR) [22], depression, and CSF biomarkers (t-tau, p-tau, amyloid β) [23,24]. Also, other demographic variables were registered (gender, age, educational level, treatment, comorbidity, alcohol consumption, tobacco consumption) (see Table 1). As expected, the CSF biomarkers levels (t-tau, p-tau, β amyloid) and the neuropsychological tests showed significant differences between groups. Age and gender showed statistically significant differences between groups. The control group showed higher educational studies compared to the case group. No significant differences were observed regarding treatment, comorbidity, depression, and tobacco or alcohol consumption.

### 2.2. Determination of Oxidation Biomarkers in the Urine Samples

**Figure 2:**
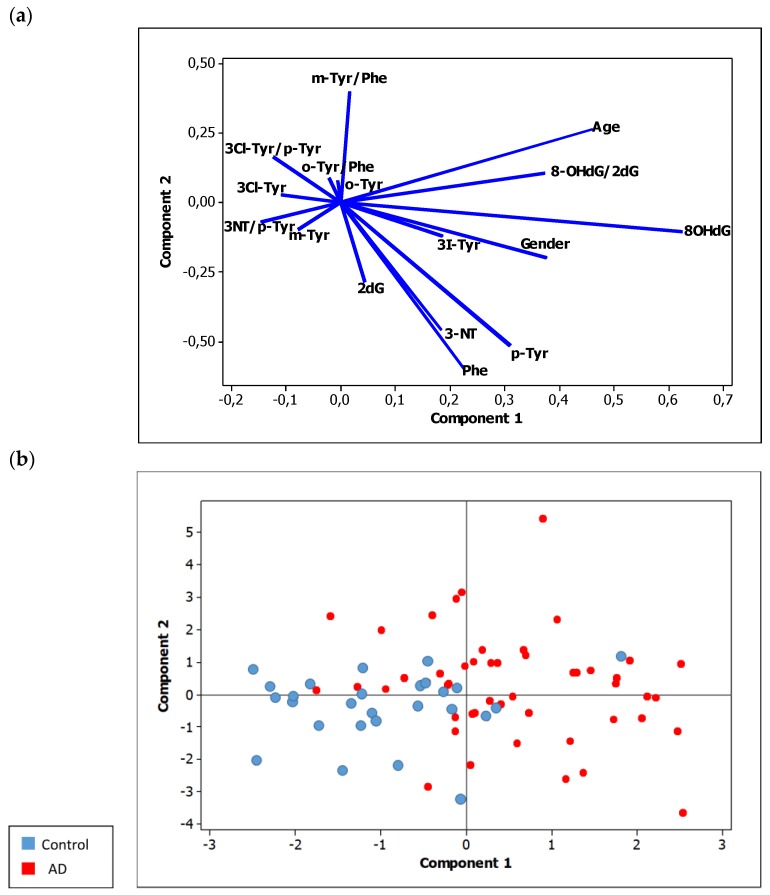
PLS model. (**a**) Loading plot (predictor variables). 3-NT: 3-nitrotyrosine, 8-OHdG: 8-hidroxyi-2′-deoxyguanosine, 3-Cl-Tyr: 3-clorotyrosine, o-Tyr: ortho-tyrosine, m-Tyr: meta-tyrosine, 2dG: 2′-deoxyguanosine, Phe: Phenylalanine; (**b**) Scores plot (participants samples).

The PLS model allowed a preliminary study of correlations between predictor variables (biomarkers levels) and the response variable (group), as well as good discrimination between participants. In this model, 16 independent variables (nine individual biomarkers, five biomarker ratios, age, and gender) were spatially distributed in order to enhance the separation between the two participant groups (0 = control, 1 = MCI-AD). In the loading plots (Figure 2a), we observed that 8-OHdG and 3-I-Tyr levels correlated with age and gender. This suggested that these variables varied together and increased also with age. Besides, the levels of m-Tyr/Phe showed an inverse correlation with 2-dG, not explaining the differences between groups. In addition, a correlation between 3-NO_2_-Tyr, Phe, and p-Tyr was observed. The scores plot showed a satisfactory separation between the participants’ groups (Figure 2b). In this sense, the case group showed higher levels of 8-OHdG and 8-OHdG/2-dG, while the control group showed higher levels of 3-Cl-Tyr, m-Tyr, and 3-NO_2_-Tyr/p-Tyr.

**Figure 3:**
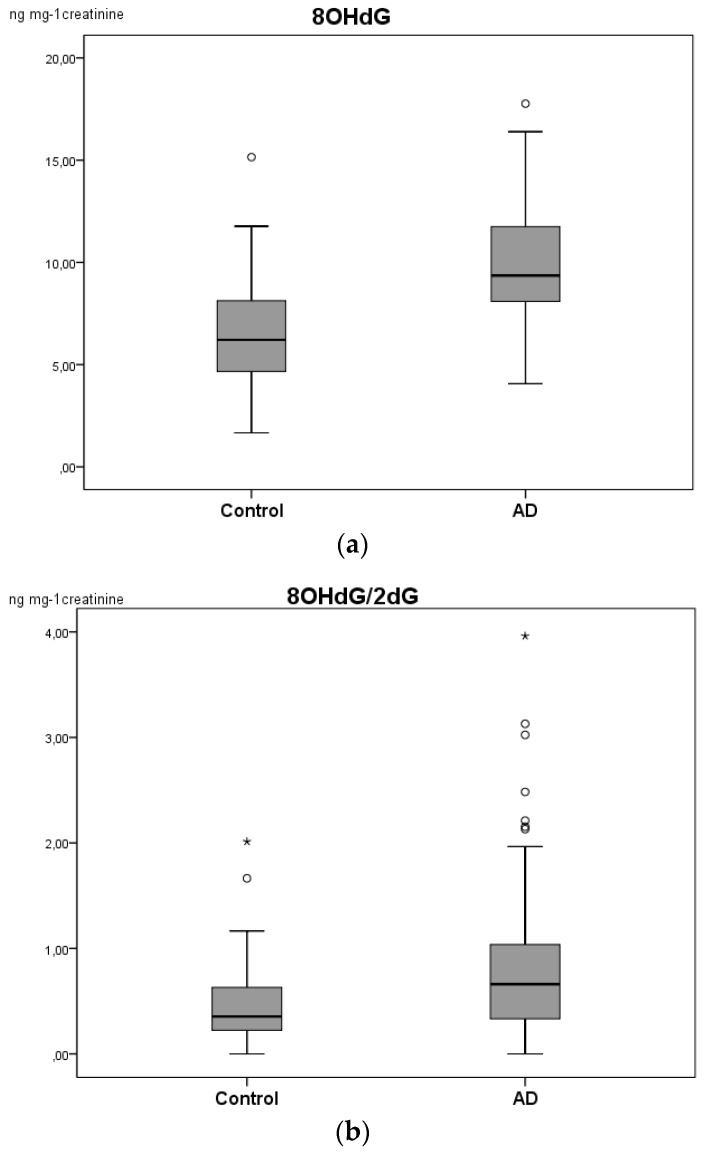
Boxplot for (**a**) 8-OHdG and (**b**) 8-OHdG/2dG ratio in the urine samples of control individuals and MCI-AD (mild cognitive impairment-Alzheimer’s disease) patients. * Points at a greater distance from the median than 1.5 times the IQR.

In order to confirm the results obtained from the multivariable analysis of biomarkers panel, a univariate statistical analysis was carried out with SPSS statistics. The levels of each analyte were compared between the groups using the Mann–Whitney test. Urine levels of 8-OHdG were higher among the MCI-AD patients compared to the healthy participants (Table 2), as well as the ratio 8-OHdG/2dG (Figure 3, Table 2). Thus, the results showed that the medians for 8-OHdG (*p* = 0.000) and 8-OHdG/2dG (*p* = 0.019) were significantly different between control and MCI-AD groups (Table 2, Figure 3). No statistically significant differences were observed for the other compounds. In addition, 8-OHdG, 3-NO_2_-Tyr, p-Tyr, and Phe showed statistically significant differences between male and female groups, and the 8-OHdG/2-dG ratio correlated with age. For this reason, we included age and gender as co-variables in the multivariate model.

**Figure 4:**
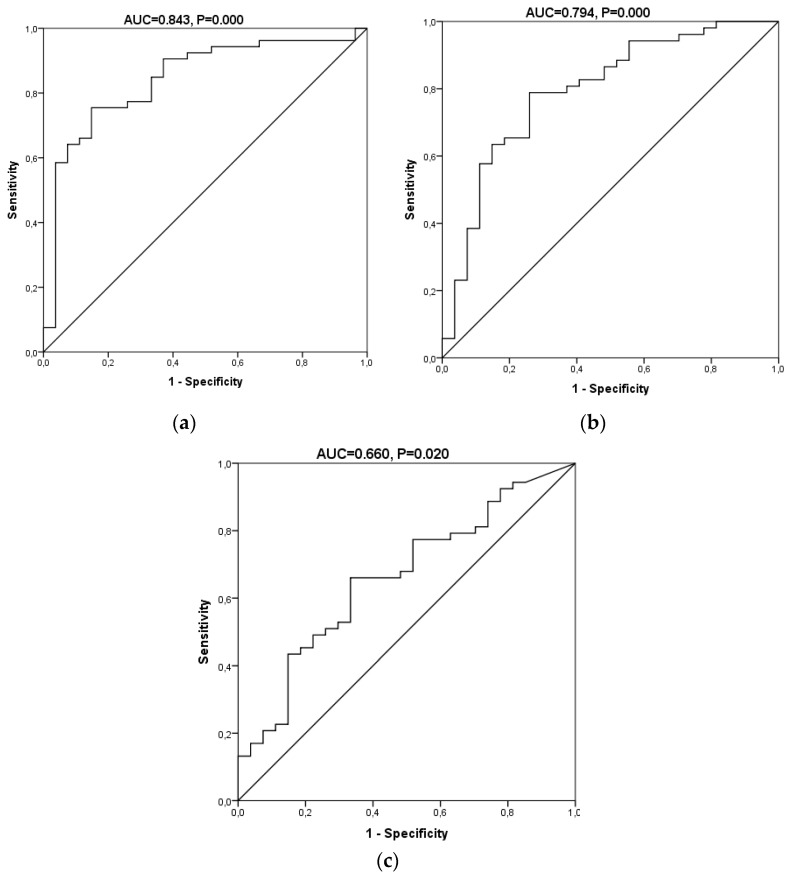
Receiver operating characteristic (ROC) curves for the oxidative biomarkers in the urine samples. (**a**) A model including all the protein and DNA oxidation products. (**b**) A model with 8-OHdG. (**c**) A model with 8-OHdG/2dG.

We performed a receiver operating characteristic (ROC) curve analysis to estimate the diagnostic potential of this panel of oxidative biomarkers in AD (biomarkers panel). Taking into account all the analytes, gender, and age, the area under the curve (AUC) was 0.843 (0.750–0.936) (*p* = 0.000) (Figure 4). The diagnostic indices calculated for this diagnostic test are summarized in Table 3. It showed a sensitivity of 78.4% and a specificity of 85.2%. Its positive predictive (PPV) and negative predictive values (NPV) were 90.9% and 67.6%, respectively, and its positive likelihood ratio (LR+) and negative likelihood ratio (LR−) were 5.29 and 0.25, showing an odds ratio of 20.91. In addition, ROC curves for 8-OHdG and the ratio 8-OHdG/2dG was performed, showing an AUC of 0.794 (0.687–0.902) and 0.66 (0.536–0.785), respectively. For 8-OHdG, the sensitivity was 77.4% and specificity 74.1, and PPV and NPV were 85.4 and 62.5, showing an odds ratio of 9.76. Finally, the clinical indices obtained for 8-OHdG/2-dG were 66.0 and 66.7 for sensitivity and specificity, PPV of 79.5, NPV of 50.0, and the odds ratio was 3.89. The better diagnostic indices obtained from the panel of biomarkers could be explained by the large and complementary information provided by the different biomarkers.

## 3. Discussion

There is strong evidence of oxidative stress being involved in the pathogenesis of AD since the brain is particularly vulnerable to the oxygen radicals and reactive oxygen species (ROS). In addition, some studies showed that these biochemical differences could be observed in peripheral tissue, such as blood samples [25,26], and urine, saliva, and hair samples [27]. Specifically, some studies have found increased oxidation product levels (inflammatory markers, oxidized proteins, lipid peroxides, glycated proteins) in CSF, serum, and plasma samples from AD patients compared to age-related controls [1,7,8,9,10,15,16,17,18,19]. However, there is an increased need to find a set of specific and reliable markers that can be measured in peripheral fluids, such as urine or plasma, and maybe potentially used as markers for AD diagnosis, even though these findings may not be a reflection of the amyloidosis state.

The present study was conducted to determine oxidative peripheral biomarkers and include elderly patients with MCI-AD and normal elderly subjects, in order to determine if oxidative products of proteins and DNA can be used as early peripheral AD biomarkers. Our results showed a significant difference between groups for 8-OHdG and the ratio 8-OHdG/2-dG (Table 2). In fact, 8-OHdG is a significant marker of DNA oxidative damage, and the ratio 8-OHdG/2-dG reflects the oxidation as a function of the not hydroxylated free base 2’-deoxyguanosine (2-dG). Therefore, the ratio 8-OHdG/2-dG could assess the oxidative damage to the DNA independently of the efficacy of the DNA repairing mechanisms [14].

However, our results did not show any significant differences, regarding tyrosine nitration (3-NO_2_-Tyr/p-Tyr), oxidation (m-Tyr/Phe and o-Tyr/Phe), and chlorination (3-Cl-Tyr/p-Tyr). Although previous studies have observed an increase in 3-NO_2_-Tyr and other protein product levels in AD plasma and CSF samples, our data did not corroborate these previous results [15,28]. This might be due to the fact of using urine samples and that the nitration product levels are below the limit of detection. Similarly, it may occur for 3-Cl-Tyr. As regarding the protein oxidation products, the levels of o-Tyr and m-Tyr in urine were similar between the two participant groups. Therefore, our study showed that elevated oxidative products were associated with AD and demonstrated that these products could be measured in urine. Specifically, the levels of 8-OHdG and the 8-OHdG/2-dG might be used as potential biomarkers of oxidative damage to DNA for early AD diagnosis from peripheral samples. Actually, these new biomarkers could show diagnostic or prognostic value in AD, or they could allow advancing in the knowledge of neurodegeneration mechanisms. Regarding specificity, the biomarkers 8-OHdG and 8-OHdG/2-dG showed correlations with the standard CSF biomarkers for AD diagnosis (β-amyloid, p-tau, t-tau). Nevertheless, further studies are required to see whether these biomarkers distinguish different forms of neurodegenerative disorders (frontotemporal dementia, dementia with Lewy Bodies, vascular dementia, Parkinson’s disease, multiple sclerosis, etc.).

To conclude, the main scientific finding indicated that urine from MCI-AD subjects showed higher oxidation levels than urine from control subjects. Also, the developed model that integrated the various products of protein and DNA oxidation showed in general suitable diagnostic indices constituting a useful non-invasive diagnosis tool for early AD, representing a more complete reflection of oxidation in patients with AD. Previous studies also indicated that multivariate models that included different analytes showed better accuracy in multifactorial pathologies diagnosis, such as AD [29,30]. Nevertheless, further studies, including an external cohort of AD patients, as well as patients with other neurodegenerative diseases, in which oxidative stress could also play an important role, are required to validate these early AD diagnosis models.

## 4. Material and Methods

### 4.1. Study Design and Participants

The eligible participants for this prospective observational study were people between 50 and 80 years old, who suffered from MCI due to AD (MCI-AD) recruited from out-patient neurology (case group), and healthy individuals (control group). The study was carried out in the Neurology Unit of the University and Polytechnic Hospital La Fe, Valencia (Spain). The diagnosis criteria for MCI-AD in this study were based on recent revisions of the National Institute on Aging-Alzheimer’s Association (NIA-AA) [31,32]. According to this, the diagnosis in this study was based on CSF biomarkers, neuropsychological testing, and structural neuroimaging by nuclear magnetic resonance (NMR) or computerized axial tomography (CAT) applied to all participants, as in previous studies [33].

Specifically, eligibility criteria for the case group (MCI-AD) included cognitive impairment, without impaired daily living activities, as shown by neuropsychological test (CDR, altered RBANS-DM), and with positive biomarkers for AD (neuroimaging, CSF p-tau 181, CFS β-amyloid 1-42); and for the control group, people with absence of cognitive disturbances (normal cognition and normal function as shown by complete neuropsychological testing), and with negative biomarkers for AD (neuroimaging, CSF biomarkers). Participants not accomplishing all the conditions defined for each group or with hydrocephalous, high grade of vascular sub-corticoid brain pathology, and other brain lesions (neuroimaging) were excluded. Also, patients with other known neurological impairments, or major psychiatric disorders, as well as patients with moderate to severe dementia, major sensory impairment, or an invalidating previous pathology were excluded from the study.

The neuropsychological battery used for this study consisted of neuropsychological and functional assessment (mini-mental state examination (MMSE), repeatable battery for the assessment of neuropsychological status (RBANS) with scores according to five domains (immediate memory-RBANS.IM, visuospatial/constructional-RBANS.V/C, language-RBANS.L, attention-RBANS.A, delayed memory-RBANS.DM), functionality assessment questionnaire (FAQ), clinical dementia rating (CDR)) [20,21,22,34].

The study protocol (2016/0257, November 2016) was approved by the Ethics Committee (CEIC) at the Health Research Institute La Fe (Valencia), and informed consent was obtained from all the participants. They were recruited between January 2017 and December 2017, and classified into control (*n* = 27) and case (*n* = 53) groups. The patients’ characteristics are summarized in Table 1.

### 4.2. Materials and Reagents

Standards of phenylalanine (Phe), para-tyrosine (p-Tyr), ortho-tyrosine (o-Tyr), meta-tyrosine (m-Tyr), 3-nitrotyrosine (3-NO2-Tyr), 3-chlorotyrosine (3-Cl-Tyr), 3-iodotyrosine (3-I-Tyr), 8-oxo-2-deoxyguanosine (8-OHdG), 2-deoxyguanosine (2-dG) (96% *w*/*w* purity) were obtained from Sigma-Aldrich (St. Louis, MO, USA). Deuterated phenylalanine (Phe-D_5_) with a 98% atom D enrichment was purchased from CDN Isotopes (Pointe-Claire, QC, Canada), and it was used as an internal standard. Water was Milli-Q grade (18.2 MV) from a Millipore purification system. Acetonitrile (ACN) (LC-MS grade), methanol (MeOH) (LC-MS grade), and formic acid (analytical grade) were purchased from Sigma Aldrich Química SA (Madrid, Spain).

Creatinine was quantified in urine samples with the enzyme immunoassay (EIA) MicroVue Creatinine kit purchased from Quidel Corporation (Athens, GA, USA) and the spectrophotometer Halo Led 96 from Dynamica Scientific Ltd (Livingston, United Kingdom).

### 4.3. Sample Collection and Treatment

For this study, the urine samples were collected from MCI-AD patients (*n* = 53) and age-related controls (*n* = 27), using sterile pots. They were centrifuged, aliquoted, and stored at −80 °C until they were processed. During preparation, the urine samples were thawed on ice to minimize the biological degradation of analytes. Then, they were homogenized by shaking on a Vortex mixer during 20 s and centrifuged at 4 °C and 7500 rpm (UVAT Bio, Valencia, Spain) for 10 min to remove large particles. A total of 200 µL aliquot of supernatants were acidified with 20 µL of H_2_O (0.5% *v*/*v* HCOOH), and 95 µL of the acidified samples and 5 µL of internal standard solution ((Phe-D_5_ and 2dG-13C,15, N_2_, 10 µmol/L each one) were loaded on 96-well plates and analyzed randomly by an ultra-performance liquid chromatography-tandem mass spectrometry (UPLC-MS/MS) analysis.

Creatinine concentrations were determined in urine samples with the MicroVue Creatinine EIA kit following the protocol recommended. Briefly, 50 µL of the diluted samples were incubated with the color solution (from the kit) during 30 ± 2.5 min at 18–28 °C. Then, the optical density was measured at 490 nm (Halo Led 96).

### 4.4. Stock, Working, and Standard Solutions

Individual stock solutions of Phe (10 mmol L^−1^), p-Tyr (10 mmol L^−1^), m-Tyr (2 mmol L^−1^), o-Tyr (2 mmol L^−1^), 3-NO_2_-Tyr (2 mmol L^−1^), 3-Cl-Tyr (2 mmol L^−1^), 3-I-Tyr (1 mmol L^−1^), 2-dG (2 mmol L^−1^), 8-OHdG (1 mmol L^−1^), and Phe-D_5_ (1 mmol L^−1^) were prepared in H_2_O (0.1% *v*/*v* HCOOH). Aliquots were obtained from the stock solutions and stored at −20 °C.

Mix solution was prepared by diluting the stock solutions in H2O (0.1 % *v*/*v* HCOOH) and kept at −20 °C. The mix solution went through a single freeze and thaw cycle. Standard solutions were prepared by serial dilution of the mix solution in the following concentrations intervals: 8-OHdG (0.2–2500 nmol L^−1^), p-Tyr (0.2–2000 µmol L^−1^), m-Tyr (0.2–2500 nmol L^−1^), o-Tyr (1–10,000 nmol L^−1^), Phe (0.2–20,000 µmol L^−1^), 3-NO₂-Tyr (1–10,000 nmol L^−1^), 3-Cl-Tyr (2–20,000 nmol L^−1^), 2-dG (1–10,000 nmol L^−1^), 3-I-Tyr (2–20,000 nmol L^−1^). Concentration ranges were determined during a pre-validation study that measured concentrations found in urine samples.

### 4.5. UPLC-MS/MS Analysis

UPLC-MS/MS analysis was carried out by means of the analytical method previously described by Carretero et al. [27]. The chromatographic system used consisted of a Waters Acquity UPLC-Xevo TQD system (Milford, MA, USA), and the analytical column was an Acquity UPLC HSS T3 1.8 μm (2.1 × 100 mm) also from Waters. Briefly, mobile phases A and B consisted of deionized water and ACN, respectively, both with 0.1% of formic acid. The flow rate was 0.3 mL/min, and the run time was 7 min. Analytes were determined by electrospray ionization (ESI) using multiple reaction monitoring (MRM) in positive mode. More instrumental parameters are described by Carretero et al. [35].

### 4.6. Statistical Analysis

Univariate statistical analyses were performed using IBM^®^ SPSS^®^ Statistics version 20.0 (SPSS, Inc., Chicago, IL, USA). All values were expressed as a number of cases (*n*) and percentage (%), mean ± standard deviation (SD), or median (interquartile range, IQR). *p*-values of <0.05 were considered statistically significant. Mann–Whitney test was used to compare medians between 2 groups (case and control). A chi-square test was employed to compare categorical variables (percentage, *n*).

Multivariate analysis was carried out using the Minitab 15.1.20.0 software (Minitab, Inc., State College, Pennsylvania, USA). We constructed a multivariable regression model of partial least squares (PLS), based on the oxidative stress biomarkers levels to discriminate between healthy and MCI-AD participants that also includes age and gender. The analysis was performed with 16 independent variables (predictors) and 1 dependent variable (response). All the variables were centered, and the predictors were auto-scaled. The model was validated by cross-validation, and the predictive ability was reflected by the percentage of validation explained variance.

To evaluate the diagnostic potential of this panel of biomarkers, we constructed a receiver operating characteristic (ROC) curve and calculated the area under the curve (AUC, 95% confidence interval (CI)). The cut-off values in the prediction of AD were established as the highest sum of specificity and sensitivity for each marker in the ROC curve. Then, we calculated the diagnostic indices for the biomarker panel (sensitivity, specificity, positive predictive value (PPV), negative predictive value (NPV), positive likelihood ratio (LR+), and negative likelihood ratio (LR−)).

## Figures and Tables

**Table 1:** Clinical and demographic characteristics.

Gender (female) (*n* (%))		**MCI-AD (*n* = 53)**	**Control (*n* = 27)**	***p*-value**
		32 (60.4%)	10 (37%)	0.048
Age (years) (median (IQR))		70.50 (68.25–74.00)	66.00 (62.00–70.00)	0.003
Educational level (*n* (%))	Primary	30 (57%)	6 (24%)	0.026
	Secondary	11 (20%)	10 (36%)	
	Academic	12 (23%)	11 (40%)	
Treatment (*n* (%))	None	20 (38%)	13 (46%)	0.374
	Psychotropic drug	7 (13%)	3 (12%)	
	Others	26 (49%)	11 (42%)	
Comorbidity (*n* (%))	None	21 (41%)	11 (42%)	0.223
	Dyslipidemia	20 (37%)	6 (21%)	
	Hypertension	12 (22%)	10 (37%)	
Alcohol consumption (yes, *n* (%))		4 (8%)	6 (24%)	0.065
Tobacco consumption (yes, *n* (%))		18 (34%)	12 (44%)	0.399
Depression (yes, *n* (%))		7 (12%)	2 (8%)	0.599
GDS (median (IQR))		8.00 (3.00–11.50)	4.00 (1.00–8.00)	0.006
β-Amyloid (median (IQR)) (pg mL^−1^) *		600.50 (450.75–727.75)	1197.00 (1124.50–1423.50)	0.000
t-Tau (median (IQR)) (pg mL^−1^) *		572.50 (372.00–818.00)	196.00 (141.00–326.00)	0.000
p-Tau (median (IQR)) (pg mL^−1^) *		85.00 (69.25–107.75)	48.00 (34.00–68.50)	0.000
Temporal atrophy (yes, *n* (%))		40 (76%)	3 (12%)	0.000
CDR	0.0 (*n* (%))	9 (16%)	26 (96.3%)	0.000
	0.5 (*n* (%))	29 (55%)	1 (3.7%)	
	1.0 (*n* (%))	13 (25%)	0	
	2.0 (*n* (%))	2 (4%)	0	
MMSE (median (IQR))		24.00 (20.00–26.00)	30.00 (28.00–30.00)	0.000
RBANS.IM (median (IQR))		61 (44–71)	90 (81–106)	0.000
RBANS.VC (scores, mean ± SD)		78 (65–89)	96 (84–112)	0.000
RBANS.L (scores, mean ± SD)		60 (54–83.5)	92 (87–96)	0.000
RBANS.A (scores, mean ± SD)		60 (53–79)	100 (85–112)	0.000
RBANS.DM (scores, mean ± SD)		44 (40–58)	100 (88–106)	0.000
FAQ (scores, mean ± SD)		6 (2.5–12)	0 (0–0)	0.000

IQR: inter-quartile range; GDS: Geriatric Depression Scale; * Biochemical determinations (β-amyloid, t-Tau, p-Tau) were carried out by Innotest Elisa kit (Fujirebio Diagnostics, Ghent, Belgium) using a fully automated system (Lumipulse G, Fujirebio). Impaired levels: β-amyloid < 700 pg mL^−1^, t-Tau > 400 pg mL^−1^, p-Tau > 85 pg mL^−1^.

**Table 2:** Analyte concentrations found in urine samples from both groups of participants.

Analyte	Median (IQR) ng mg^−1^ Creatinine		*p*-value (Mann–Whitney Test)
	MCI-AD (*n* = 53)	Control (*n* = 27)	
3-I-Tyr	2.08 (0.87–3.18)	1.82 (1.01–2.59)	0.412
8-OHdG	9.46 (8.09–12.02)	6.21 (4.44–8.41)	0.000 *
2-dG	14.84 (6.21–29.57)	12.89 (5.26–22.07)	0.479
3-NO_2_-Tyr	36.89 (25.86–74.58)	43.78 (24.87–63.00)	0.835
3-Cl-Tyr	-	-	-
o-Tyr	-	-	-
m-Tyr	4.54 (1.97–6.79)	5.53 (2.68–8.06)	0.593
p-Tyr	7441.69 (5318.80–12904.17)	6574.32 (4755.32–11780.08)	0.292
Phe	76349.94 (54047.28–121009.87)	82324.57 (61530.76–95751.69)	0.875
mTyr/Phe ^a^	0.000057 (0.000027–0.000099)	0.000059 (0.000030–0.000077)	0.725
oTyr/Phe ^a^	-	-	-
3-NO_2_-Tyr/ pTyr ^a^	0.0050 (0.0037–0.0069)	0.0050 (0.0038–0.0087)	0.593
3-Cl-Tyr/ pTyr ^a^	-	-	-
8-OHdG/ 2-dG ^a^	0.6963 (0.3347–1.0796)	0.3811 (0.2883–0.7072)	0.019 *

^a^ Ratios; * *p* < 0.05.

**Table 3:** Diagnostic indices for the biomarkers panel.

Indexes	Biomarkers Panel	8-OHdG	8-OHdG/2-dG
AUC (95% CI)	0.843 (0.750–0.936)	0.794 (0.687–0.902)	0.66 (0.536–0.785)
Sensitivity (%, 95% CI)	78.4 (65.4–87.5)	77.4 (64.5–86.5)	66.0 (52.6–77.3)
Specificity (%, 95% CI)	85.2 (67.5–94.1)	74.1 (55.3–86.8)	66.7 (47.8–81.4)
PPV (%, 95% CI)	90.9 (78.8–96.4)	85.4 (72.8–92.8)	79.5 (65.5–88.8)
NPV (%, 95% CI)	67.6 (50.8–80.9)	62.5 (45.3–77.1)	50.0 (34.5–65.5)
LR+ (95% CI)	5.29 (2.12–13.23)	2.98 (1.55–5.74)	1.98 (1.12–3.49)
LR− (95% CI)	0.25 (0.15–0.43)	0.31 (0.18–0.53)	0.51 (0.33–0.78)
DOR (95% CI)	20.91 (5.97–73.28)	9.76 (3.33–28.59)	3.89 (1.46–10.38)

## Abbreviations

AD	Alzheimer’s Disease
CSF	Cerebrospinal fluid
DOR	Diagnostic odds ratio
ELISA	Enzyme-linked immunosorbent assay
IQR	Interquartile range
LR−	Negative likelihood ratio
LR+	Positive likelihood ratio
MCI	Mild cognitive impairment
MS	Mass spectrometry
NFT	Neurofibrillary tangles
NPV	Negative predictive value
HPLC	High-performance liquid chromatography
EC	Electrochemical detection
DNPH	2,4-dinitrophenylhydrazide
SD	Standard deviation
PLS	Partial least squares
PPV	Positive predictive value
